# Development of a New Application-Based Chewing Efficiency Test (Mini Dental Assessment) and Its Evaluation by Nursing Staff in Geriatric Care: A Pilot Study

**DOI:** 10.3390/ijerph182211889

**Published:** 2021-11-12

**Authors:** Alexander Schmidt, Maximiliane Amelie Schlenz, Clara Sophie Gäbler, Steffen Schlee, Bernd Wöstmann

**Affiliations:** 1 Dental Clinic, Department of Prosthodontics, Justus Liebig University Giessen, 35392 Giessen, Germany; alexander.schmidt@dentist.med.uni-giessen.de (A.S.); clara.s.gaebler@dentist.med.uni-giessen.de (C.S.G.); bernd.woestmann@dentist.med.uni-giessen.de (B.W.); 2 Department of Geriatrics, County Hospital Frankenberg, 35066 Frankenberg, Germany; steffenschlee@t-online.de

**Keywords:** mastication, geriatric assessment, nutrition assessment, nursing assessment, dentistry, oral health care service delivery, real world data on dentistry, geriatric oral health, patient care, analogue-digital conversion

## Abstract

The increasing average life expectancy worldwide results in an elderly population with significant health care needs. However, dental care is often not a focus of care. It is well known that oral and overall health are directly related. Therefore, the Mini Dental Assessment (MDA) was developed to provide a simple analysis of oral health status, although it is currently only available in paper form, with all associated drawbacks, from illegible writing to the inability to quickly search the collected forms. This study aimed to develop a digital application (app) for mobile devices that can overcome the problems associated with paper forms. After the digital MDA was developed, its usability was evaluated by nurses, a questionnaire was answered, and it was compared to the analog MDA with patients in a pilot study. The usability of the app (System Usability Scale) was 95.18 ± 4.26, representing a very high usability. Furthermore, this app showed good clinical applicability. The results also showed that the digital MDA was accepted by nurses in their daily routine and was preferred to the analog MDA. A follow-up study with a higher number of subjects is highly recommended.

## 1. Introduction

The increasing life expectancy and its associated demographic transition have led to a growing elderly population in many countries. Numerous people require clinical or home care; thus, the number of geriatric patients is also increasing [1,2]. However, dental care is often out of the focus of nursing care; a shortage of nurses, an increase in general diseases up to multimorbidity, and the high cost of healthcare systems has reduced the time available for care for each patient [3]. This is aggravated by the difficulty in assessing patients’ dental status in a nursing home environment for non-dentists. In addition, patients in nursing homes or clinical institutions are often unable to visit dental practitioners or clinics. Thus, oral health status is rarely monitored [4]. This is complicated by the so-called “paradox of aging”, which describes how patients become more satisfied with their situation despite the objectively declining health with increasing age [5]. For example, as patients’ age, they have lower expectations regarding their dentures. This can lead to an “underreporting”, making problems with existing dentures often noticed or treated too late or not at all [6]. In a previous investigation, we observed that poorly rated dentures correlate with a tendency toward poorer chewing efficiency [7], which applies especially to elderly people.

Taken together, patients with objectively poor oral health are often subject to the opinion that they are doing well. Since they had no complaints, they are not referred to a dentist [8]. Clearly, there is a distinct need for an easy and reliable method for nursing staff and geriatrics to identify patients who are subjectively satisfied with their oral situation but objectively have a high need for dental treatment. The first approach was the analog Mini Dental Assessment (MDA), which was developed for an easy analysis of oral health status [8]. Caregivers conduct this assessment using a chewing efficiency test with carrots and ask a patient about the age of his most recent dental prosthesis and the time since the last visit to the dentist. From these data, a score can be calculated, which indicates whether a visit to the dentist is necessary or advisable, or if a regular checkup will do.

MDA has already been validated by dental professionals in comparison with other assessments and has been successfully applied to patients [8]. However, MDA is currently only available in paper form, making it extremely time-intensive and impractical to collect data. The feedback provided via the paper forms also varies greatly in quality, as it depends entirely on the oral health knowledge (or lack thereof) of the healthcare provider. Therefore, an analog-digital conversion of MDA in paper form to a new digital assessment application (app)-based MDA is urgently needed. In other areas of dentistry, such as the manufacture of complete dentures, digitization has already been successfully used to improve the care of elderly patients [9]. In times of nursing crisis and overburdened health systems, digitalization might be a solution to maintain the quality of life of elderly people [10].

The present study aimed to develop a new app-based MDA for mobile devices based on the existing analog MDA, and to examine its practicability and acceptance by non-dental nursing staff in a pilot study. Furthermore, a questionnaire survey of nursing staff was conducted to collect feedback on their experience with the two MDAs (analog and digital). Moreover, a dentist examined all patients, subsequent to the MDA evaluation by nursing staff, to validate the results of the MDAs. 

The following null hypothesis was tested: nursing staff will prefer the new digital app-based MDA and that it will yield valid results for estimating patients’ need for dental care.

## 2. Materials and Methods

### 2.1. Setup of the Mini Dental Assessment (MDA) 

Before applying the analog MDA that was first described by Wöstmann et al. [8], patients were asked if they had toothache or painful pressure spots. If the patient answers affirmative to the question about pain, it is recommended for the patient to make an immediate appointment with the dentist. If the patient does not have pain, MDA can be initiated. The user will be guided throughout the complete MDA by an instruction in paper form. The template of the analog MDA can be downloaded free of charge from the website of the dental clinic of the Justus Liebig University Giessen [11].

First, a chewing efficiency test was performed. Therefore, a customary carrot must be cut into a standardized cube (20 × 20 × 10 mm). Then, the cube of the carrot was placed in the patient’s mouth and the patient was asked to chew the carrot as small as possible for 45 s without swallowing the bolus. At the end of the chewing process, the bolus is collected on a flat surface (e.g., petri dish), and excess saliva is removed before visual inspection. This was followed by visual assessment of the degree of comminution of the carrot using a 6-point ranking scale (1 = excellent, 2 = good, 3 = medium, 4 = moderate, 5 = poor, 6 = impossible). The respective result of the degree of chewing is assigned as numerical values from 10 to 60; this numerical value is subsequently noted on the analog MDA template. 

Subsequently, the patients were asked about the time point of their last dental visit. If the last visit was less than a year ago, a numerical value of 0 was noted, and when the time was unknown, a numerical value of 10 was given. The numerical value was then multiplied by a factor of three, and the value was noted. 

Finally, the patient was asked about the age of their most recent dentures. If only natural teeth were present and the patient had no dentures, a value of 0 was noted. If there were no teeth or dentures, a value of 10 was given. The value of the latest dentures in years was then noted. 

**Figure 1:**
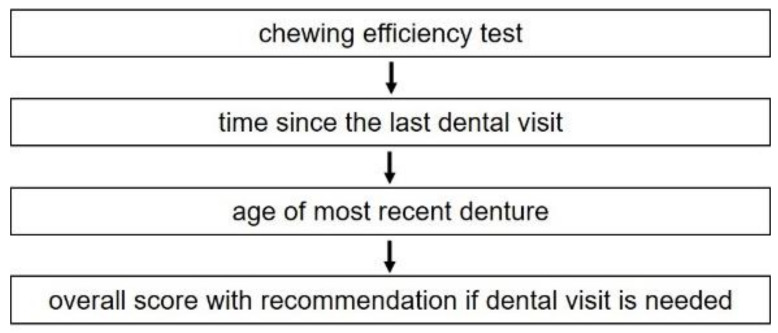
Flow scheme of the different steps of the MDA to acquire an overall score for recommendation if a dental visit is required.

Numerical values from the chewing efficiency test and treatment history are summarized. The resulting numerical value is noted and gives an overall score. With a score of 10–30, no dental treatment is needed, and only routine checkup is recommended. If the score is between 31–60, patients should be checked by a dentist. If the score is over 60, dental treatment is strongly recommended. The flow scheme in Figure 1 shows the different steps of the MDA.

### 2.2. Interobserver Agreement

Prior to the app development, an interobserver agreement test between different users for the chewing efficiency test of the MDA was conducted. Therefore, 12 dentists, 10 nurses, and 10 non-professional dental or medical personnel assessed 100 photos of chewed carrots and assigned them to one of the six comminution grades of the MDA. The results of all groups were assessed by an independent dentist and compared with each other.

### 2.3. App Development for the Digital MDA

The application software was programmed using the MIT App Inventor 2 (Massachusetts Institute of Technology, Cambridge, MA, USA), which allows the storage of the MDA app on the computer as an Android package file. Windows 10 (Microsoft Corporation, Redmond, WA, USA) was used as the operating system and Mozilla Firefox 78.0.2 (Mozilla Corporation, Mountain View, CA, USA) as the browser for MDA app development. Galaxy Tab A 2016 (10 inch) tablets from Samsung (Android version 9, Samsung Electronics, Seoul, South Korea) were used as mobile devices. The software was installed on tablets via data transfer. However, for further use, no data connection to the tablet was necessary as the software ran completely autonomously.

**Figure 2:**
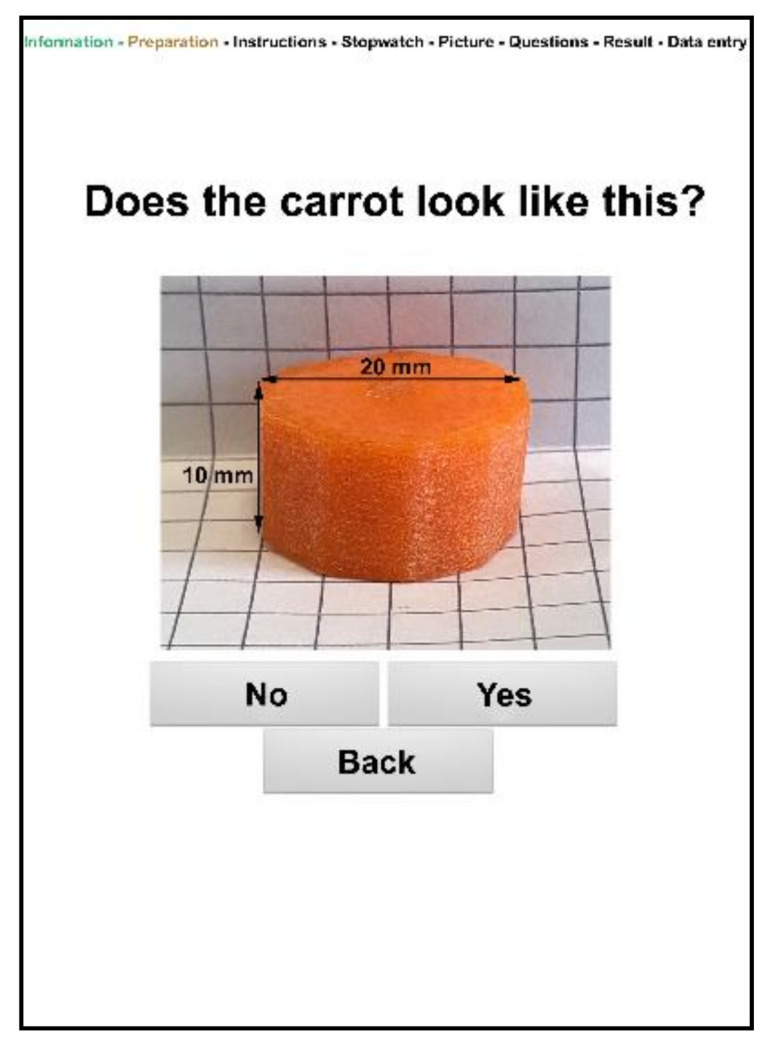
Screenshot of the digital MDA control question regarding the correct size of the carrot cube (20 × 20 × 10 mm) for standardized investigation.

The instructions for preparing the carrot cube, practical application, and questions regarding the patient’s dental treatment history of the analog MDA were transferred to the digital MDA. Furthermore, control questions were implemented. For example, the user will be asked if the carrot has the correct size (Figure 2). 

**Figure 3:**
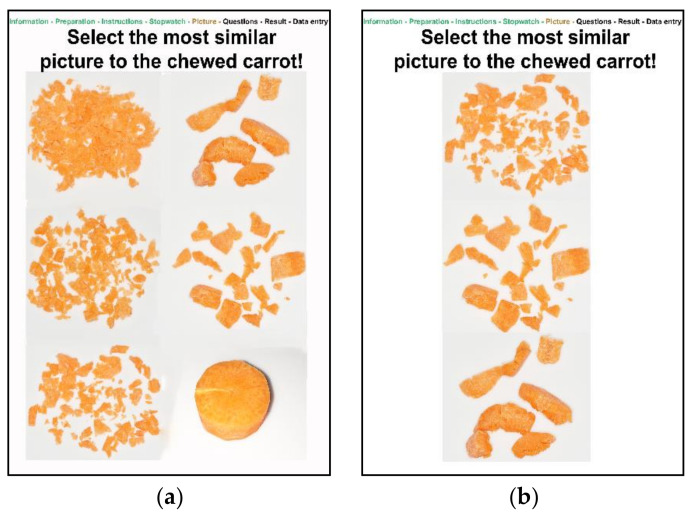
Screenshot of the digital MDA control questions with two-step decision making; (**a**) carrots of different degrees of comminution; (**b**) carrots of different degrees of comminution.

For the visual assessment of the degree of comminution of the carrot using a 6-point ranking scale (1 = excellent, 2 = good, 3 = medium, 4 = moderate, 5 = poor, 6 = impossible), a two-step decision for verification was implemented. First, the user displays six pictures of different degrees of comminution and selects the one that looks most similar to the chewed carrots of the patient (Figure 3a). Afterwards, the user is asked to choose between three pictures with the next higher and lower degrees of comminution (Figure 3b). If the user decides to choose another picture as selected before, the score for the chewing efficiency test will be automatically reduced by 5 points.

**Figure 4:**
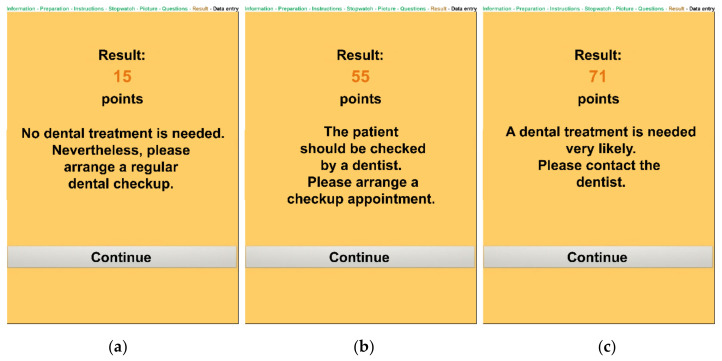
Screenshot of the digital MDA displaying the three possible results of the assessment: (**a**) 10–30 points, (**b**) 31–60 points, (**c**) >60 points.

An integrated timer displays 45 s for chewing the carrot cube and reminds the user when time is over that the patient has to disgorge the carrot bolus. Furthermore, the software automatically calculates the score of the digital MDA, and, at the end, the user obtains the results of the assessment with an explanation of what to do with the patient regarding dental treatment (Figure 4). 

### 2.4. Usability Test of the Digital MDA

To test the usability of the digital MDA, a pre-test was conducted with a representative group of 14 participants (8 female, 6 male; age, 41.1 ± 13.1 years) not previously involved in the MDA app development. The usability test took place in a quiet setting, and no time limit was set. To accurately evaluate the subjects’ verbal assessments, written records were prepared by the investigator. For the usability test, the subjects were asked to perform predetermined tasks using the MDA app. For this purpose, three patient cases were given on the basis of a photo of the chewing efficiency test, and information about the patient’s dental treatment history was provided. The participants were also asked to identify the data of a patient previously entered into the database by the investigator. Participants were instructed to express their thoughts aloud during task completion according to the think-aloud method [12]. Subsequently, participants completed a System Usability Scale (SUS) [13]. The results were digitized, and the selected scale position was converted into a numerical value. The SUS score was also calculated for each participant, and the overall mean with standard deviation was determined. The test subjects’ written statements and suggestions for improvement regarding the MDA app were categorized thematically. Before clinical data collection, all suggestions for improvement were implemented and integrated into the app.

### 2.5. Pilot Study Using Analog and Digital MDA in Daily Routine

To investigate the practicability and acceptance by non-dental nursing staff in the daily routine of geriatric care, a pilot study was conducted. Therefore, for a first clinical application, five randomly selected nurses (5 female; age 35.6 ± 11.5 years) of the Department of Geriatrics of the County Hospital Frankenberg without any dental background were asked to apply the analog and digital MDA to their patients. The patients recruited for the pilot study were aged 71–91 years and were either fully dentated or had fixed or removable dentures. Participation in this study was voluntary, and patients addicted to medication, drugs, or alcohol, suffering from a tumor, being on parenteral nutrition, undergoing radiotherapy, suffering from dysphagia or craniomandibular dysfunction, had undergone orthodontic treatment in the last three years, with pathological clinical symptoms, or suffering from salivary dysfunction (xerostomia) were excluded.

The study was conducted in accordance with the guidelines of the World Medical Association Declaration of Helsinki and approved by the local ethics committee of the Justus Liebig University Giessen (Ref. No. 291/20). Furthermore, the study was registered in the German Register of Clinical Trials (DRKS00027742; CONSORT Checklist). All participants were informed of the background of this study and signed informed consent forms. A dentist monitored the study to standardize the experimental procedures.

All five nurses completed the analog and digital MDA without assistance. To avoid transferring the MDA results from one to another, different patients for analog and digital MDA were examined. Therefore, a total of 10 applications (five analog and five digital) of MDA were conducted. As a reference, two experienced dentists, who were not involved in the study, determined the chewing efficiency of the investigated patients to obtain reference values, and disagreements were discussed.

### 2.6. Questionnaire

Immediately after conducting the analog and digital MDA, the five nurses were asked to fill out the questionnaire (Table S1), which was designed in cooperation with the Teaching Evaluation Center of the Justus Liebig University Giessen. In addition to evaluative statements regarding the attitude towards MDA in general and analog and digital MDA in specific, the applicability in daily routine were enquired. Participants could agree or disagree with the statements using a 5-point Likert scale [14]. Finally, the demographic questions were asked. Abstention of answers were allowed, and questionnaires were evaluated anonymously.

### 2.7. Statistical Analysis

Statistical analysis was conducted using IBM statistics version 26 (IBM Germany GmbH, Ehningen, Germany), with an alpha error level set at 5%. For the interobserver agreement, the interclass correlation (ICC) was calculated as two-way random, absolute agreement, single rater. In addition, the mean values of the assessment of 100 images per subject were determined.

The results of the SUS were digitized, and the selected scale position was converted into a numerical value. The SUS score, as well as the mean and standard deviation, were calculated for each respondent individually. The subjects’ written statements and suggestions for the improvement of the MDA app were thematically categorized and evaluated. The deviations of the subject data from the reference value were calculated to evaluate the data of the analog and digital MDA. 

Results of questionnaires are presented as the mean and standard deviation.

## 3. Results

### 3.1. Interobserver Agreement

The ICC results describing the interobserver agreement are displayed in Table 1.

The results showed only slight deviations in ICC mean values and therefore, only a slight deviation in the subjects’ estimation of the degree of shredding of the respective carrots. The different groups also did not differ significantly from each other. Furthermore, data show that dentists achieve only slightly better agreement values than nurses and dental or medical non-professionals.

### 3.2. System Usability Scale (SUS)

The result of the SUS questionnaire was 95.18 ± 4.26 (mean ± standard deviation), which conforms to an excellent (A) rating score [13].

### 3.3. Practicability and Acceptance of Analog and Digital MDA

First, the deviations between the analog and digital MDA from the reference values were analyzed. Table 2 displays the results of the “chewing efficiency test”. 

Half of the scores of the chewing efficiency test were consistent with the reference values, whereas in three cases, the deviation of the analog MDA from the reference values was −10 points. This means that the chewing efficiency was evaluated in these three cases; one grade of the six-point-scale was too good as the actual situation was. However, in two cases, the chewing efficiency in the digital MDA was rated too high. The odd values of −5 and −15 points are the results of the two-step decision-making system of the digital MDA, which is not available for the analog MDA. The results for the “time since the last dental visit” were compared (Table 3). Similar to the chewing efficiency test, half of the answers regarding the “time since the last visit” was consistent with the reference values. However, the deviations in the other half were not more than 2 years.

In the analog MDA in four of five subjects, the last visit to the dentist was not correctly transformed by the nursing staff into the correct MDA point values, which was clearly other the anticipated. 

In Table 4, the deviations regarding the “age of the most recent dentures” are presented. In one case of digital MDA, the age of the most recent denture was not recorded correctly.

Finally, the overall scores between the analog and digital MDA to the reference results were analyzed (Table 5). A slightly higher number of cases of digital MDA were found. However, the analog and digital MDA showed good agreement with the reference values.

### 3.4. Questionnaire

All nurses who participated in this pilot study stated that they preferred using the digital instead of the analog MDA. However, two nurses can imagine using both analog and digital MDAs in daily routine, whereas the other three nurses only wanted to use the digital MDA. Furthermore, all nurses considered MDA to be useful, especially for the detection of malnutrition (Table 6 and Table 7). 

## 4. Discussion

The basic aim of this study was to develop a digital application (app) for mobile devices to determine the oral health status of patients. This should help to overcome the problems associated with paper forms. After the app was developed, the usability of the app was assessed using the System Usability Scale. It showed very high usability, which allowed the app to be compared to the analog MDA in a pilot study in a clinical setup on patients.

Whether in home care by relatives or in outpatient care in nursing homes, there is often a lack of knowledge about oral health care. A patient’s dental prosthesis is also often not at the forefront of a caregiver’s mind [3].

Investigations in nursing homes have observed that the oral hygiene and dental care of residents have often been neglected in the past or even did not occur at all [15]. The results show that in 46% of retirement homes no regular inspection of dental and oral health took place. In 23% of the homes, occasional checks were dine, and 31% of regular checks were completed. Sometimes, the last visit to a dentist was 8 years ago or even longer [16]. This is confirmed by the DMS V study, in which 60% of elderly patients in need of care can no longer independently make an appointment with a dentist or visit a dental practice [4]. Finally, due to the connection between a person’s susceptibility to general illnesses and lack of oral health, there is increasing recognition of the need for improvement in monitoring the oral health and dental care of elderly and care-needy patients. 

At this point, a tool that empowers the nursing staff to evaluate dental status is necessary. Nurses are required to be experts in the broad field of medicine and geriatrics, and to be professionals in all areas. However, it is often very difficult to assess the oral health status and existing prostheses of patients by inspection. Therefore, interdisciplinary assessments were established within the field of nursing, especially in geriatrics, to be able to reliably assess a patient’s existing health situation, such as the Mini Nutritional Assessment (MNA) [17,18] or the Mini Mental Status (MMS) [19].

To determine the oral health status and its associated chewing efficiency, chewing function tests were performed. These tests are defined as adequately crushing food within a certain number of chewing cycles [16,20,21,22]. As early as 1950, Manly and Branley [23] showed that sieving the crushed food bolus is a useful method for testing chewing efficiency, and this method is still used today [21,24]. Chewing efficiency can be influenced by age, sex [25,26,27], and the number of occluding tooth pairs [27,28,29]. Patients with less than 20 teeth have worse chewing efficiency than those with more than 20 teeth [24].

Currently, two test methods have been used to detect chewing ability. First, a patient is asked to chew chewing gums of different colors, and the degree of mixing was measured [30,31,32]. In the second test, patients were given fruit gums of different colors and asked to chew them [29,33,34,35,36,37]. The greater the mixing, the better the chewing efficiency. In addition to subjective visual inspection [29,34,35], the degree of mixing can be determined by computer analysis [37,38,39,40].

However, a major problem with chewing gum tests is that test results can be evaluated only through a technically demanding process that requires a scanner and is more complex for laymen to perform. In addition, fruit gums have different colors and degrees of hardness and have to be sorted by size after chewing.

Clearly, an assessment that is easier to implement is necessary. The oral health status and chewing efficiency should be tested, as this allows conclusions to be drawn about any necessary visit to the dentist. Therefore, MDA was developed in 2011.

To investigate the applicability, reliability, and validity of the individual test procedures, these were examined and compared in previous investigations [8,41]. It could be observed that the MDA is a suitable instrument in assessing whether a dental treatment need exists. Overall, the MDA seems to be a suitable instrument to help nursing home staff assess the need for nursing home residents to make an appointment with a dentist.

The results of the interobserver agreements of the present study showed that there were no significant differences in the assessment of the degree of chewing of the carrot for different individuals with different prior medical knowledge. This is consistent with previous studies [8,41] and indicates a wide range of applications for different investigators.

However, MDA was only available in analog form. For easy handling and integration into the clinical routine, a digital form using an app for mobile devices was required in addition to the analog form.

A major advantage of the app, in addition to storage and paperless application, is the possibility of implementing a second step to verify the degree of crushing of the carrots. This allows a more detailed result compared with the analog form. In addition, possible errors that can occur when adding the individual values within the analog MDA are excluded by an automated calculation within the app and although avoid simple calculation and transformation errors as seen in Table 4. This was confirmed by the results of the present study, while there were no transfer errors when using the digital MDA. In addition, a stopwatch is integrated into the app; thus, additional timekeeping is not necessary. Compared to the digital MDA, most of the deviations from the correct result in the analog MDA are based on calculation errors or transmission errors of the entire summation.

A possible source of error is the question of the “last visit to the dentist” and the “age of the most recent dental prosthesis”, which can lead to misstatements, especially in patients with dementia in both MDA types (analog and digital). This is a clear limitation of the present study. However, digitizing the process enables the oral health status of a patient in need of care to be determined quickly and efficiently. Especially in the digital MDA, it was possible to integrate a database into the MDA app. This makes it possible to observe the patient individually over a certain period of time and determine the necessary dental visits or deterioration of the oral health condition. This also makes it easier for the investigator to assess any misreporting by the patient. Compared to analog MDA, this is much easier to perform, and the data are anonymized.

In addition, digitizing the MDA makes it possible to collect data over a broad population, as the data are to be stored anonymously. This collective database makes it possible to assess the oral health status of the greater population, and potentially help identify steps that can be taken to improve the oral health of the elderly, the fastest growing part of our society.

Based on the findings of this clinical pilot study, it can be concluded that the MDA app is very useful for detecting a patient’s current treatment needs, even in the home or nursing environment. As the question of the use of the MDA by dental and non-dental staff could be answered, the question now arises as to whether the MDA can also be used in the area of home care for relatives. Therefore, a follow-up study with a higher number of participants is highly recommended.

## 5. Conclusions

The results of the present study showed a high level of agreement in the performance of MDA, regardless of prior medical knowledge. Furthermore, a digital MDA can be developed in the form of an app, which allows an easy and user-friendly application. The present study also suggests that MDA can be used in both analog and digital applications in everyday clinical practice. In addition, the digital MDA was accepted by the nurses in daily routine and was preferred to the analog MDA.

## Figures and Tables

**Table 1:** Results of the intraclass coefficient (ICC).

	ICC	95% Confidence Interval	
		Lower Limit	Upper Limit
Dentists (n = 12)	0.936	0.917	0.953
Nurses (n = 10)	0.931	0.909	0.949
Non-professionals (n = 10)	0.932	0.911	0.950

**Table 2:** Results for the “chewing efficiency test”—deviation between the MDA and reference values for the analog (n = 5) and digital (n = 5) MDA. The absence of deviation from the reference value is shown in bold type.

Chewing Efficiency Test		MDA		Total [n]
		Analog [n]	Digital [n]	
deviation between MDA and reference values [points]	−20	0	0	0
	−15	/	1	1
	−10	3	0	3
	−5	/	1	1
	**0**	**3**	**2**	**5**
total [n]		5	5	10

**Table 3:** Results for the “time since the last dental visit”—deviation between the MDA and reference values for the analog (n = 5) and digital (n = 5) MDA. The absence of deviation from the reference value is shown in bold type.

Time Since Last Visit to the Dentist with Triple Rating		MDA		Total [n]
		Analog [n]	Digital [n]	
deviation between MDA and reference values [years]	**0**	**1**	**4**	**5**
	1	3	0	4
	2	1	1	1
total [n]		5	5	10

**Table 4:** Results for the “age of most recent denture”—deviation between the MDA and reference values for the analog (n = 5) and digital (n = 5) MDA. The absence of deviation from the reference value is shown in bold type.

Age of Most Recent Denture		MDA		Total [n]
		Analog [n]	Digital [n]	
deviation between MDA and reference values [years]	−10	0	1	1
	**0**	**5**	**4**	**9**
total [n]		5	5	10

**Table 5:** Results for the “overall score”—deviation between the MDA and reference values for the analog (n = 5) and digital (n = 5) MDA. The absence of deviation from the reference value is shown in bold type.

Overall Score		MDA		Total [n]
		Analog [n]	Digital [n]	
deviation between MDA and reference values [points]	−3	1	1	2
	**0**	**3**	**4**	**7**
	+3	1	0	1
total [n]		5	5	10

**Table 6:** Items and descriptive statistics of questionnaire regarding attitude towards MDA in general.

Item Description	Nurses (n = 5)	
	Mean	SD
**What do you consider of importance using the MDA?**		
Easy comprehension ^a^	4.6	0.6
Easy application ^a^	4.6	0.6
**Pleasant execution for the investigated patient ^a^**	4	1
Fast application ^a^	4.4	0.9
**To what extend do you agree to the following statements?**		
**Digital nursing assessments simplifies everyday care. ^a^**	4.4	0.9
**There should be more apps especially for nursing staff. ^a^**	4.4	0.9

^a^ type of answer: 1 = strongly disagree, 5 = strongly agree.

**Table 7:** Items and descriptive statistics of questionnaire regarding the use of analog and digital MDA answered by nurses (n = 5).

Analog MDA		Overall Assessment	Digital MDA	
Mean	SD		Mean	SD
3.6	0.6	Easy comprehension ^a^	4.8	0.5
3.8	0.8	Helpful tool to carry out the MDA ^a^	4.6	0.6
3.8	0.8	Good applicability with patients ^a^	4.4	0.6
1.8	1.3	Easy documentation of chewing efficiancy ^a^	4.8	0.5
1.8	1.3	I would use the MDA in daily routine. ^a^	5	0

^a^ type of answer: 1 = strongly disagree, 5 = strongly agree.

## Data Availability

The datasets of this article are available from the corresponding author on a reasonable request.

## Supplementary Materials

The following are available online at https://www.mdpi.com/article/10.3390/ijerph182211889/s1, Table S1: Mini Dental Assessment (MDA) Questionnaire.
