# Supplementary material for: Development of a New Application-Based Chewing Efficiency Test (Mini Dental Assessment) and Its Evaluation by Nursing Staff in Geriatric Care: A Pilot Study
Source: Int J Environ Res Public Health. 2021 Nov 12;18(22):11889. doi: 10.3390/ijerph182211889 (PMC8625330; doi:10.3390/ijerph182211889)
Supplement: Supplementary file 1 [file ijerph-18-11889-s001.zip › ijerph-1430107-supplementary.pdf]

Mini Dental Assessment (MDA) Questionnaire

Dear nurses,

You have kindly agreed to participate in this study to develop an app-based chewing efficiency test (Mini Dental Assessment app).

Patients in need of care often receive dental examinations and treatments too late. From a dental perspective, these patients are often underserved. Clearly, there is a distinct need for an easy and reliable method for nursing staff and geriatrics to identify patients who objectively have a high need for dental treatment. The MDA is a simple screening test to determine the dental treatment needs of patients. This allows dental treatment of these patients in a timely manner.

We would like to ask you to read the questionnaire thoroughly.

Please use this questionnaire to compare the analog version (form) with the digital version (app).

All responses will be kept strictly confidential in accordance with the Data Protection Regulation.

Thank you for your support!

1.Please check only one box per answer option!

	**strongly disagree……………………..……………………...agree strongly**
**What do you consider of importance using the MDA?**	
Easy comprehension	
Easy application	
Pleasant execution for the investigated patient	
Fast application	
**To what extend do you agree to the following statements?**	
Digital nursing assessments simplifies everyday care.	
There should be more apps especially for nursing staff.	

2.Please check only one box per answer option!

**The digital version (app)…**	**strongly disagree……………………..……………………......….agree strongly**
…was easy to comprehend.	
…was helpful tool to carry out the MDA.	
…was good for applicability with patients.	
…was easy for documentation of chewing efficiency.	
…would I use for daily routine.	

**The analog version (form)…**	**strongly disagree……………………..……………………......….agree strongly**
…was easy to comprehend.	
…was helpful tool to carry out the MDA.	
…was good for applicability with patients.	
…was easy for documentation of chewing efficiency.	
…would I use for daily routine.	

3.Which version of the MDA did you prefer?

- □ The analog version (form).
- □ The digital version (app).

4.Can you imagine using MDA in daily routine?

□ Yes, both the analog version (form) and the digital version (app) can be integrated into everyday care routine.
□ Yes, but only the analog version (form) can be integrated into everyday care routine.
□ Yes, but only the digital version (app) can be integrated into everyday care routine.
□ No, neither the analog version (form) nor the digital version (app) can be integrated into everyday care routine, because …

5.Please fill in your gender and age!

Gender:
□ female
□ male
□ diverse
Age: ____ years
